# Outcomes of a brief mental health and resilience pilot intervention for young women in an urban slum in Dehradun, North India: a quasi-experimental study

**DOI:** 10.1186/s13033-018-0226-y

**Published:** 2018-08-18

**Authors:** K. Mathias, A. Pandey, G. Armstrong, P. Diksha, M. Kermode

**Affiliations:** 1 Emmanuel Hospital Association, New Delhi, India; 2 Austin Health, Heidelberg, VIC Australia; 3 Centre for Mental Health, Melbourne School of Population and Global Health, University of Melbourne, Melbourne, Australia; 4 University of Melbourne, Melbourne, Australia; 5 Nossal Institute for Global Health, University of Melbourne, Melbourne, Australia; 6 Present Address: Landour Community Hospital, Mussoorie, Uttarakhand 248179 India

**Keywords:** Youth mental health, Resilience, Depression, Anxiety, Peer-based intervention, India, Poverty

## Abstract

**Background:**

Mental illness is a leading cause of the disease burden among young people. Poor mental health is linked to childhood adversity such as gender inequality, poverty and low educational attainment. Psycho-social assets in adolescents can moderate these impacts and be strengthened. The aim of this study was to assess the effectiveness of a brief mental health and resilience intervention among disadvantaged young women in urban North India.

**Methods:**

We used an uncontrolled repeated measures design to evaluate the effectiveness of the 15-module mental health and resilience curriculum among young women residing in a slum in Dehradun, Uttarakhand. Standardised psychometric assessments were done to assess outcomes of the intervention at three time-points: pre-intervention (T1), post-intervention (T2), and 8-months post-intervention (T3), covering domains of self-efficacy, resilience, anxiety, depression and gender attitudes.

**Results:**

Young women completing the intervention (n = 106) had all left school before 10th class. A statistically significant improvement in all psychometric measures was found at T2. These improvements were sustained at T3 in the areas of anxiety, depression and gender equality attitudes, while the measures of resilience and self-efficacy had declined to baseline.

**Conclusions:**

This intervention delivered by community-based peers among highly disadvantaged young women can lead to sustained improvements in anxiety and depression and attitudes to gender equality. While other studies in LMIC have shown increased adolescent resilience through peer-led curriculums, this study demonstrates improvements in mental health and gender attitudes can endure 8-months post-intervention. This low-cost, brief intervention can improve mental health resiliency and self-efficacy among disadvantaged young people. Further research should explore how to bring sustained improvements in resilience.

## Background

There are more than 260 million young people in India, greater than the total combined populations of France, Germany, Canada and the United Kingdom. Mental distress is a leading cause of health-related burden for India’s young people [1], which is aggravated by a high prevalence of socio-economic adversity such as physical and sexual abuse, poverty, low education and gender inequality [1, 2].

Poverty and mental ill health are inter-related through mediating pathways that include access to political recognition and economic power [3]. Low educational status and associated low literacy is another significant form of adversity that leads to poorer health outcomes and reduced opportunities for developing protective social and cognitive skills, leading to increased risk for mental distress [4]. Conversely, higher levels of education have been linked with better mental health [5].

India is one of the most gender unequal countries in the world [6], meaning Indian young women have multiple pathways to disadvantage that cumulatively and differentially impact wellbeing [7]. In this part of the world they are likely to be economically and socially vulnerable with poorer health, education and livelihood outcomes compared to young men [7–9]. Young women are also at greater risk of mental distress, which is related to hegemonic masculinity that values males and disadvantages females [8].

Resilience refers to the process of, capacity for, or outcome of successful adaptation despite challenging or threatening circumstances [10]. Adolescence is a critical time for building protective psycho-social characteristics that support resilience such as effective planning skills, persistence, empathy, self-regard and kindness. These assets can moderate the relationship between experiences of adversity such as poverty or gender exclusion, and the occurrence of mental distress among young people [11]. Such psycho-social assets can be targeted and developed by resilience, youth development or social-emotional learning interventions which have been shown to improve attitudes towards self and others (self-efficacy), social and emotional skills, behaviour, physical health and academic performance [11, 12].

There has been very little research on mental health, resilience and protective factors among young people in low and middle-income countries (LMICs) [13], and few evaluations of the effectiveness of indigenously developed interventions [2, 14]. A recent randomized controlled trial assessing a school-based 5-month programme to build resilience among girls from disadvantaged backgrounds in the Indian state of Bihar found improved emotional resilience, self-efficacy, social and psychological well-being in the intervention group compared to the controls [15]. This study did not evaluate outcomes beyond the end of the intervention [15].

The aim of this study was to evaluate the effectiveness of a 15-week structured and peer-led youth development and resilience pilot intervention (titled *‘Nae Disha’* or ‘New Pathways’) that aimed to improve resilience, reduce psychological distress, and promote well-being and positive gender attitudes. In this paper, we hypothesise that the *Nae Disha* intervention will improve emotional resilience and self-efficacy and reduce anxiety and depression among young people, and that these changes will endure over time. To our knowledge this is the first evaluation of the effectiveness of an intervention to reduced anxiety and depression, promote attitudes of gender equality, self efficacy and resilience among highly disadvantaged young women outside of a school setting in India.

## Methods

### Study design

Our study utilised an uncontrolled repeated measures design to evaluate the effectiveness of the *Nae Disha* intervention where peer-facilitated groups met weekly for 15 weeks covering a structured curriculum designed to improve youth resilience, mental health and wellbeing and equal gender attitudes. The curriculum is described in detail in Table 1 below.

**Table 1:** Summary of modules in the *Nae Disha* curriculum

Theme	Modules	Sample of content
Self-identity and esteem	Modules 1–3	Module 1: Welcome, group games and rules Module 2: Self-esteem—discussions on how we are worthy and valued Module 3: Understanding our differences—difference between sex and gender, gender roles
Identifying and managing emotions	Module 4	Module 4: Managing strong emotions—use emoticon game to discuss different emotions—sharing about recent event of feeling anger—ways to manage anger and frustration
Mental health	Modules 5–7	Module 5: Sharing of understanding of mental health and common mental illness. Use a spectrum to plot own mental wellbeing and discussions on a case story of risk and protective factors Module 6: Discuss signs and symptoms of severe mental illness and ways to support those affected Module 7: Coping with tension—coping strategies
Communication skills	Modules 9,10	Module 9: Learning to communicate confidently—open sharing of passive, confident and aggressive styles of communication. Introduce and practice use of “I” messages and practice active listening skills Module 10: Stopping bullying—recognising and responding
Relationship skills and bouncing back –forgiveness	Module 8, 12	Module 8: Bouncing back—share experiences of failure and not meeting parental/peer expectations. Re-introduce six steps for problem solving Module 12: Relationships for life
Self-care and drawing boundaries	Modules 13, 14	Module 13: Saying ‘No’ to alcohol and drugs Module 14: Protecting ourselves—open discussion about sexual abuse/harassment, legal setting and community support structures. Pairs prepare response on different scenarios and share
Planning skills and citizenship	Modules 11, 15	Module 11: Collective decision and planning for a joint project—“I can create change” Module 15: Each group develops a small collective action for change in community action

### Setting

This intervention was conducted in an urban area known as *Purani Basti* (Old Slum), on the Rispana Rao river, a seasonal drain in north-east Dehradun, Uttarakhand. A 2013 house-to-house survey in this basti covering 1660 households found that 10.3% of houses had open drains, and only 58.3% of the population were literate [16]. The population comprises migrants from different North Indian states with significant representation of people from oppressed castes (including Balmiki and Scheduled Caste members) and an oppressed group of Sikhs from Punjab. The current study was implemented by Burans, a community mental health partnership project led by the Emmanuel Hospital Association [17] with the Uttarakhand Community Health Global Network. Burans had collaborated in the delivery of the Corstone Girls First curriculum to 600 young people attending government schools in different locations of the district in the previous year, and had received training and support from the Corstone India team to deliver the Girls First programme [12, 18].

### Sample selection

Through house to house visits in *Purani Basti*, Burans field staff sought to identify all young women aged 12–24 years who were not attending school and who consented to participate in the *Nae Disha* programme. A total of 110 participants were identified and other residents confirmed this was likely a 100% sample of young women fitting these inclusion criteria. Of these 106 young women completed the intervention i.e. a participation rate of 96.4%. These participants were formed into eight groups of 12–15 members based on geographic proximity to each other.

### Intervention

#### Intervention content

The *Nae Disha* was developed by the first author and others in the Emmanuel Hospital Association integrating theoretical underpinnings of Ottawa charter for health promotion [19], and a youth resilience approach that seeks to facilitate development of psycho-social assets to promote youth citizenship and positive youth development [20–22]. Table 1 summarises curriculum content.

#### Intervention delivery

Group facilitators were locally recruited young women aged 20–30 years who expressed enthusiasm to work in youth resilience. Additional criteria for recruitment were that they should be female, and have completed 12th class. They were given 5 days of training that addressed group facilitation skills as well as curriculum content and a 2-day refresher training of after completion of eight modules. Young women were formed into eight groups with 12–15 participants per group that met in the home of one of the group members by agreement with their household members, for 15 consecutive weeks. They facilitated *Nae Disha* in pairs. A team leader supported facilitators with planning, reporting and stepped in if a peer facilitator was unable to attend. A *Nae Disha* field coordinator supported all aspects of the intervention delivery and performed monitoring and support.

#### Intervention participation, quality and fidelity

Group attendance records showed the average number of sessions attended was 13.8 out of a possible 15. Fidelity and quality were assessed by observation of peer facilitators by the project coordinator.

### Data collection

Burans field staff trained in use of the psychometric tools, data recording and management, and ethical research, were supervised and supported by a research assistant and the first author. Data was collected in July 2015, October 2015 and June 2016 requiring two 45-minute sessions each time. After first completing demographic information related to housing, parental education, schooling and other factors linked to socio-economic status, young people completed the psycho-metric assessments. Those with limited literacy were assisted by a staff member. Four participants dropped out of the programme as they had found paid work and were unable to attend the group meeting (their data are excluded from the analysis) leaving a total of 106 young women who completed the programme.

### Assessment measures

To evaluate the intervention standardised psychometric tools (also outlined below) were administered to participants at three time-points: pre-intervention (T1), post-intervention (T2), and follow-up (8-months post- intervention) (T3). All outcomes were self-reported. Standardised psycho-metric scales were selected based on their previous use in similar populations, sensitivity to similar interventions, being short and simple to administer and being either free or low cost. They were the same measures used in a large randomised controlled trial assessing girls’ resilience conducted in Bihar [18] who gave permission for their Hindi translations of these tools to be used in this study.

The scales were as follows: the Connor-Davidson resilience scale (CD-RISC) uses 25 items on a 5-point Likert scale to measure coping ability and resilience [23]. The Schwarzer’s General Self-Efficacy Scale is built on ten-items, was developed for use in diverse cultures, has been validated in Asia [24], and is positively correlated with depression, anxiety and optimism [24, 25]. The Patient Health Questionnaire (PHQ-9) is a screening tool for depressive symptoms validated for use in India and among adolescents [18, 26]. The Generalised Anxiety Disorder scale (GAD-7) is a screening tool validated to assess anxiety symptoms in diverse settings [27]. The GEMS gender attitudes scale assessed attitudes to gender equality and covers areas such as sexual and reproductive health, sexual relations, violence and domestic [28]. The scales were originally translated into Hindi, trialled and validated among young people in India through the Corstone youth resilience programme in Bihar [12].

Higher scores in the scales for resilience, gender equality and self-efficacy denoted greater well-being, while higher scores in GAD-7 and PHQ-9 tests denoted poorer mental health and a lower score denoted greater mental health.

### Data analysis

Data were analysed in Stata version 14. Descriptive statistics were used to describe the socio-demographics of the study sample. Repeated measures ANOVAs were used to test if the scores for each of the scales changed between T1, T2 and T3. Paired t-tests were used to check whether significant changes had occurred between T1 to T2 and T1 to T3. Statistical significance was regarded as a p-value < 0.05.

### Ethics

Written informed consent was obtained from all participants in this study, with additional consent from parents of all participants who were legally minors. Study information and consent was read aloud to participants with limited literacy. Ethics consent for this study was obtained from the Emmanuel Hospital Association Institutional Ethics Committee in May 2015 (Protocol 124).

## Results

### Sociodemographic characteristics

The socio-demographic profile of the 106 participants is shown in Table 2. The mean age of participants was 16.7 years (standard deviation [SD] 2.77) and the age range of participants was 10–25 years. The mean number of people per household was 6.5 (SD 2.46, range 2–14 people) while the mean number of rooms per house was less than three. The community socio-demographic profile reveals significant markers of deprivation and poverty such as the high mean number of people per household, and the fact that nearly 80% of fathers and 96% of mothers of the young women were either unschooled or had completed fewer than 8 years of education.

**Table 2:** Socio-demographic profile of participants who completed the *Nae Disha* intervention (n = 106)

Characteristic	n (%)
Age in years (range 10–25)	
10–13	13 (12.3%)
14–17	45 (42.5%)
18–25	48 (45.3%)
Marital status	
Unmarried	101 (95.3%)
Married	5 (4.7%)
Number of people in household (range 2–14)	
Less than 4 people	19 (17.9%)
5–8 people	70 (66.0%)
Greater than 9 people	17 (16.0%)
Fathers occupation	
Self-employed	65 (61.3%)
Unskilled labourer	12 (11.3%)
Father absent from family home	22 (20.8%)
Other	7 (6.6%)
Fathers educational status	
Unschooled	62 (58.5%)
Completed up to 7 years schooling	22 (20.8%)
Completed 8 years or more of schooling	22 (20.8%)
Mothers occupation	
Self-employed	17 (16.0%)
Unskilled labourer	10 (9.4%)
Unemployed	62 (58.5%)
Mother absent from family home	4 (3.8%)
Other	13 (12.3%)
Mothers educational status	
Unschooled	72 (64.1%)
Completed up to 7 years schooling	30 (28.3%)
Completed 8 years or more of schooling	4 (3.8%)
Household assets	
Television	99 (93.4%)
Radio	29 (27.4%)
One or more mobile phones	104 (98.1%)
Vehicle (motorbike or four-wheeler)	48 (45.3%)
Ownership of agricultural land	6 (5.7%)

NB: Missing data of the four participants did not complete the intervention have been removed before calculating percentages

### Change in resilience, mental health and well-being, and gender attitudes

The mean scores for each psychometric scale are presented in Table 3, for T1, T2 and T3. The repeated measures ANOVA revealed that there was a statistically significant difference between the mean scores at pre-intervention, post-intervention and follow-up for all five scales. The paired t-tests demonstrated a statistically significant improvement between pre- and post-intervention in all scales across domains of self-efficacy, resilience, anxiety, depression and gender attitudes. The improvement in mental health and gender attitudes was sustained at follow-up, however, the improvement in the scales measuring emotional resilience and self-efficacy were not sustained.

**Table 3:** Change in mean scores on resilience, self-efficacy, anxiety, depression and gender attitudes scales between pre-intervention, post-intervention and follow-up

Scale	T1 (pre-intervention)	T2 (post-intervention)	T3 (8-month follow-up)	*p*-*value*^a^	*p*-*value*^b^ *T1*–*T2*	*p*-*value*^b^ *T1*–*T3*
CD-RISC 10	20.4 (19.2–21.6)	30.6 (29.8–31.4)	20.7 (19.5–22.0)	< 0.001	< 0.001	0.665
Schwarzer’s General Self-Efficacy	26.5 (25.2–27.7)	33.6 (32.9–34.3)	27.2 (26.1–28.3)	< 0.001	< 0.001	0.311
Patient Health Questionnaire (PHQ-9)	9.0 (8.0–10.0)	1.5 (0.7–2.3)	2.7 (2.0–3.3)	< 0.001	< 0.001	< 0.001
Generalised Anxiety Disorder (GAD-7)	8.0 (7.1–8.9)	0.7 (0.3–1.2)	2.8 (2.3–3.4)	< 0.001	< 0.001	< 0.001
Gender attitudes scale	22.0 (20.9–23.1)	26.5 (26.1–26.8)	25.6 (25.0–26.2)	< 0.001	< 0.001	< 0.001

^a^ Repeated measures ANOVA

^b^ Paired t-test

## Discussion

This study evaluates a pilot implementation of the *Nae Disha* intervention using psycho-metric measures of anxiety, depression, self-efficacy, resilience and gender attitudes at baseline, end-line and follow-up among a group of highly disadvantaged young women living in an inner-city slum in North India. The *Nae Disha* intervention significantly improved all five measures showing reduced symptoms of depression and anxiety, and greater self-efficacy and resilience and more gender-equal attitudes. These results indicate that the *Nae Disha* intervention was beneficial for these young women who had left school prematurely. The Girls First youth resilience curriculum implemented in Bihar among young women attending government schools [12, 18] did not achieve improvements in anxiety and depression measures, and it may be that the presence of two modules focused on mental health in *Nae Disha* may have contributed to the improvements in depression (PHQ-9) and anxiety (GAD-7) achieved and sustained 8 months later, in this study.

The *Nae Disha* intervention is likely to have been effective through a combination of two key mechanisms: firstly, participation in a facilitated group, which builds emotional competence, promotes peer relationships and strengthens social networks; and secondly through curriculum content promoting self-belief and resilience skills. The contribution of group membership and peer relationships to increased wellbeing and empowerment among young people has been well described internationally [29] and among women in India [30], while others have underlined the contribution of group membership and interaction to the development of social capital [31]. The curriculum content of *Nae Disha* includes several evidence-based modules describing techniques for creating behavioural change [32]. These include goal setting and planning, social support, self-belief and identity [32] which are likely to also have increased intervention effectiveness.

Psycho-metric measures of anxiety (GAD) and depression (PHQ9), and gender attitudes demonstrated improvements that endured a further 8 months. We propose mechanisms supporting gender attitude changes were triggered by the safe-space of girls-only group conversations, gender-specific role modelling of facilitators and positive attitudes towards other young women and their potential expressed by facilitators. Possible reasons for the sustained improvement in mental health could be related to the strong focus of the intervention on mental health and were perhaps less dependent on the group and were thus sustained. Improvements in areas such as self-efficacy and resilience were likely more difficult to sustain in the absence of regular group meetings.

There were also major improvements in psychometric measures of resilience (CD-RISC 10 and self-efficacy) when T1 and T2 results are compared, however over the subsequent 8 months these scores declined to T1 levels. Multiple factors linked to the ongoing environment of adversity experienced by these young women both in their homes and in the slum environment more broadly are likely to be contributing to this finding. It may be harder to change resilience than mental health because the former is a deeply-engrained personal skill honed and developed through numerous, continual life experiences, whereas mental health is ‘a state of mind’ that may be more amenable to intervention. The data in Table 2 indicate the extent of social and financial deprivation with low rates of educational attainment among men and women, and high levels of crowding. Slum dwellers occupy marginal social positions in Indian society with multiple hierarchies related to age, gender, faith tradition, caste and educational status that interact to limit the movements, social contact, social inclusion and participation of young women in any spheres of social engagement [33–35]. While this study measured mental health using PHQ9 and GAD7, given the strong association between somatic symptoms and anxiety–depression related distress among Indian women [36], somatic symptom counts would be another important outcome to include in evaluation of youth mental wellbeing interventions.

The enduring improvement in attitudes to gender equality is another important indicator of programme effectiveness. Attitudes that support gender equality are critical for empowerment and wellbeing of women [7]. The *Nae Disha* curriculum directly addressed gender differences, gender-based violence and self-protection. It also addressed gender attitudes indirectly through other modules, discussions and roles plays that addressed self-worth, confident communication, and strategies for conflict resolution. A further gender-linked component included the group community action initiative titled “I can create change”. While groups were given liberty to choose a domain for action, several groups focused on actions related to gender inequality. For example, one group facilitated a community meeting to discuss ‘education of the girl-child’ while two *Nae Disha* groups and their mothers participated in a rally promoting opportunity to complete high school education for girls. These outcomes support the approach presented by Freire [37], that seeks to develop critical consciousness and opportunity for citizenship actions, leading to empowerment, increased self-efficacy and changed gender attitudes. Other studies also demonstrate this, such as a study in South Africa which describes the contribution of citizenship and participation within a radical project for development [38] and literature which describes the contribution of citizenship within a participatory and radical politics of development [39].

This study has three main limitations: firstly, it relies on self-report measures which can have difficulties among young people who could have an inflated sense of their improvements after completion of the intervention [12]. Secondly, it is conducted with no control group so the changes observed cannot be attributed definitively to this intervention, and may have occurred secondary to another event or intervention. However, the fact that some of the improvements shown at T2 ultimately declined to the same level measured at T1 suggests that they were linked to the intervention described in this study. Thirdly, it is a study with relatively small numbers which limits the statistical power of the study.

Features of this study that increase its generalisability are the inclusion of young women of diverse religious backgrounds with significant representation of Sikh, Hindu and Muslim traditions among participants, and the fact that it was implemented in the homes of community members, so had low resource requirements. The programme demonstrated effectiveness in building resilience among vulnerable young women who were poor, had left school prematurely, and were living in an urban slum, but further research is needed to assess whether the *Nae Disha* intervention could be as effective among more advantaged young people.

The intervention was implemented with scalability in mind, with group facilitation led by local young women who had only completed high school education, and for most of whom this represented their first form of paid employment. The intervention resource is freely available for download in English and Hindi, and includes detailed instructions on facilitation to maximise accessibility for other programmes. Further studies evaluating effectiveness on a larger scale could increase external validity. Based on these study findings, this intervention would be useful for young women in communities throughout India and South Asia, and possibly for young people in other LMICs.

## Conclusions

This study is one of few studies worldwide to evaluate an intervention seeking to build mental health and resilience among young people in a deprived urban setting. It demonstrates outcomes of reduced depression and anxiety, more gender equal attitudes and improved resilience and self-efficacy among disadvantaged young women through a low-resource programme facilitated by community peers. However, as improvements in resilience and self-efficacy declined to baseline levels 8 months after the intervention, it is evident that further research is required to understand the contextual factors around young people that might support or erode improvements in resilience, as well as the form and duration of interventions needed to effect change in young people’s resilience.

This low-cost and simple intervention led to improvements in mental health and attitudes to gender equality for these young women that were sustained for 8 months after the intervention. This offers possible routes forward for building resilience and mental health the many poor, urban and young people across South Asia.

## Abbreviations

CD-RISC: Connor-Davidson Resilience scale
GEMS: gender equitable men scale
GAD-7: Generalised Anxiety Disorder scale
LMIC: low-middle income country
PHQ-9: Patient Health Questionnaire (screens for depression)
SD: standard deviation
T1, T2, T3: pre-intervention (T1), post-intervention (T2), and 8-months post- intervention (T3)
